# Controlling Electronic Coherences and the Curvature Induced by the Derivative Coupling at a Conical Intersection: A Quantum Ehrenfest (QuEh) Protocol for Reaction Path Following Application to “Channel 3” Benzene Photochemistry

**DOI:** 10.1021/acs.jpca.4c02449

**Published:** 2024-06-25

**Authors:** Graham A. Worth, Michael A. Robb

**Affiliations:** † Department of Chemistry, University College London, 20, Gordon Street, WC1H 0AJ London, U.K.; ‡ Department of Chemistry, Molecular Sciences Research Hub, Imperial College London, White City Campus, 80 Wood Lane, W12 0BZ London, U.K.

## Abstract

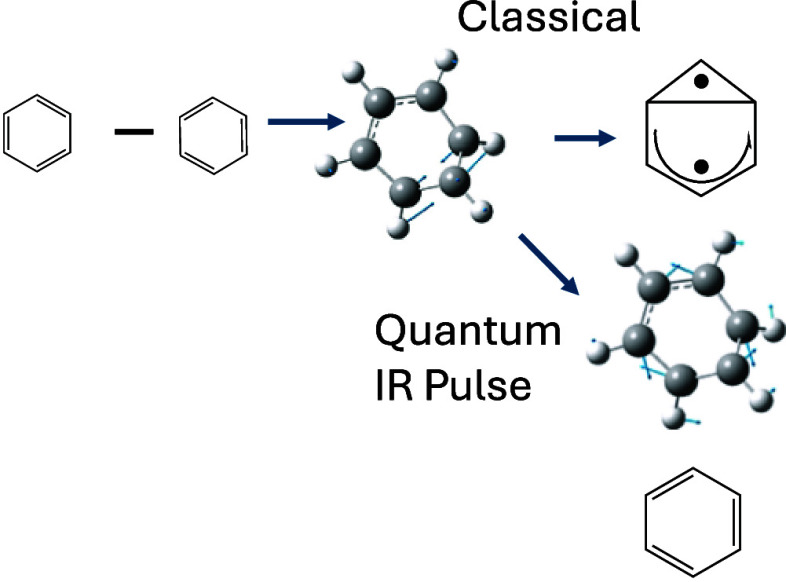

We report a protocol for the implementation of “reaction path following” from a transition state through a conical intersection, including both the path curvature induced by the derivative coupling and the corresponding induced electronic coherences. This protocol focuses on the “central” Gaussian wavepacket (initially unexcited) in the quantum Ehrenfest (QuEh) method. Like the reaction path following, the normal mode corresponding to the imaginary frequency at the transition state is given an initial momentum. The protocol is applied to the “channel 3” radiationless decay of benzene. We also demonstrate that one can enhance the effect of the derivative coupling and the electronic coherence with an IR pulse.

## 1 Introduction

In photochemistry, there are reactions where one has a transition state on the reaction path that connects the FC region and a conical intersection on the reaction path to products. The presence of such a transition state provides a dynamics bottleneck (or filter) and simplifies the mechanism. The related central concept is the reaction path. The original idea of the reaction path is due to Fukui.^1^ The connection to dynamics is to be found in the classic work of Miller Handy and Adams.^2^ A recent review that discusses both the conceptual and implementation aspects is due to Hratchian and Schlegel.^3^ The essential idea of a reaction path is associated with the path of steepest descent in mass-weighted coordinates initiated at the transition state along the normal mode with an imaginary frequency. The connection with dynamics is most easily understood from the work of Hratchian^4^ where they discuss following reaction pathways using a damped classical trajectory algorithm. The ideas just discussed are one-dimensional. In this work, we show that one can retain this one-dimensional concept but still allow for the passage through a conical intersection with the concomitant coupling with other normal modes via the derivative coupling.

**Figure 1:**
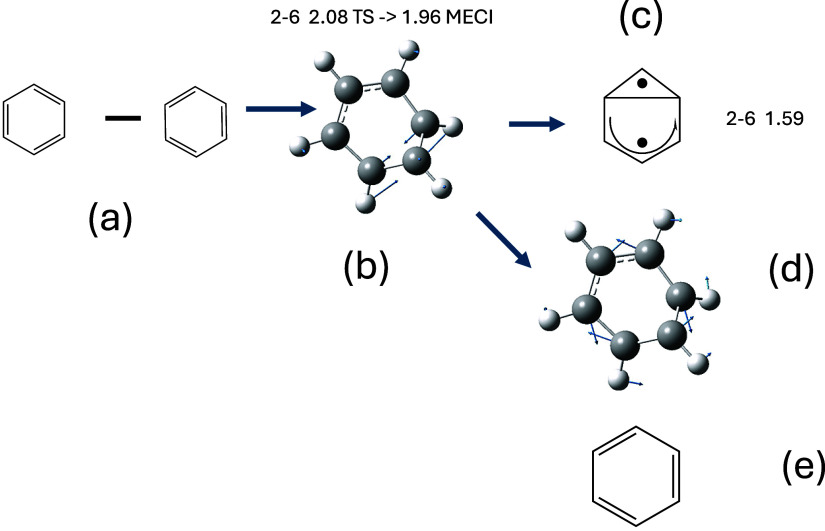
Schematic representation of the “channel 3” photochemistry of Benzene (a) S_1_ vertical excitation is to an anti-Kekulé structure (i.e., difference of two Kekulé structures, where the sum of the two Kekulé is the usual S_0_ ground state) (b) a transition state (6–2 bond length 2.08 A^0^ leading to a minimum energy conical intersection at 1.96 A^0^ (c) Prefulvene 6–2 bond length 1.59 A^0^ (note that the ground state benzvalene species is formed by coupling the two unpaired electrons) (d) a vector parallel to the derivative coupling and (e) a transient localized benzene Kekulé structure.

From a conceptual and practical point of view, the reaction path idea is useful because photochemistry can be understood in terms of a single nuclear coordinate. Of course, there are many issues such as tunneling through the barrier, etc. that are not covered by the reaction path alone. In addition, there are quantum mechanical effects such as the role of the derivative coupling at the conical intersection and the observation of electronic coherences at a conical intersection.^5,6^ It is our purpose in this article to give a protocol for the extension, using the Quantum Ehrenfest method (QuEh),^7^ of the reaction path concept within the field of nonadiabatic dynamics and attochemistry^8^ and to illustrate it with the Channel 3 benzene photochemistry, as illustrated in Figure 1. We will show that the change in the reaction pathway resulting from the derivative coupling and/or the role of electronic coherences, when probed by a nonresonant laser pulse,^9^ can also be represented within this approach. Of course, from a practical point of view, it becomes useful to start quantum dynamics from the transition state because of the time delay to reach the transition state bottleneck if one were to start at the Franck–Condon point (i.e., the geometry of the ground state).

As shown in Figure 1, we suggest that the effect of the derivative coupling on passing near the conical intersection is to rearomatize the S_1_ state of benzene (we will demonstrate this subsequently). The subject of aromaticity has been reviewed recently.^10^ In older work, we have made some studies of this effect^11,12^ in the related decay of Dewar Benzene (S_2_), and the reader is referred to recent work on photochemistry and aromaticity.^13,14^ The initial vertically excited S_1_ benzene is shown as the difference of the two Kekulé structures (S_0_ is the sum): thus obviously antiaromatic.

The second-order Ehrenfest method^15^ that we have used in other work to probe a simple reaction path,^16,17^ is different from a naïve Ehrenfest approach because the full interstate gradients are computed. These interstate gradients are the derivative couplings that arise in the off-diagonal derivative matrix elements that occur between the components of the states that are coherently mixed at a conical intersection (see Section 2 for a full discussion). This approach allows one to continue the computation of the reaction path through the conical intersection.^18^

Of course, the second-order Ehrenfest method ignores the effect of the derivative coupling between other possible trajectories/normal modes, which partly diverts the reaction path parallel to the derivative coupling (see Figure 1b–e). This idea has been reviewed recently.^19^ This effect requires the explicit computation of interaction/coupling of several trajectories and normal modes that occur in the quantum dynamics approach.^20^ Various approaches are available to transcend this limitation: One approach is to add the effect of the derivative coupling in the surface hop method.^21^ Ultimately, the only complete strategy is to treat the nuclei quantum mechanically (e.g., quantum dynamics^20^ or exact factorization^22,23^). In this work we use a related method, the quantum Ehrenfest method, (QuEh),^7^ in which the nuclei move on time-dependent potential surfaces which are determined by a solution of the time-dependent Schrödinger equation (TDSE) within a CASSCF formalism^15^ and the nuclei also satisfy the TDSE.

The reaction path on the potential energy surface of a polyatomic molecule is the steepest descent path (if mass-weighted Cartesian coordinates are used) connecting a saddle point to minima. For an N-atom system in 3d space, the 3N-6 internal coordinates can be chosen to be the reaction coordinate *s*, the arc length along the reaction path, plus (3N-7) normal coordinates that describe vibrations orthogonal to the reaction path. We now extend this idea in a representation where both nuclei and electron motion are driven by the time-dependent Schrodinger equation.^20^ The nuclear motion is computed using the variational Multiconfiguration Gaussian method^24^ for the propagation of a nuclear wavepacket as implemented in the Quantics program^25^ and the electronic motion uses the second-order Ehrenfest method.^15^ The combination results in the Quantum Ehrenfest method (QuEh).^7^

In the QuEh approach, we select 2*n* + 1 Gaussian wavepackets to describe the nuclear motion, where *n* is the number of normal modes chosen to describe the system. Each Gaussian wavepacket (gwp) is given initial coordinates by exciting a normal mode with a positive or negative displacement. The first, central, gwp (gwp 1) is not excited. Choosing which normal modes to include is delicate. As we have suggested elsewhere it is useful to recognize the isomorphism between the electronic structure (in a valence bond representation) and the selected normal modes.^26^ For benzene, we have chosen 13 normal modes to span the transition vector, the C–C stretches, and the C–C–C bends. At a transition state, one is at a critical point, so the central gwp 1 gradient is initially zero.

In a classical reaction path computation,^3^ one may give an initial displacement along the reaction path. We emulate this idea by giving an initial momentum in normal mode that corresponds to the transition vector. Thus, we start our quantum dynamics with initial momentum at a transition state geometry, and we use the normal modes of the excited state transition state structure to define our gwp basis. This protocol contrasts with the usual choice, where one starts in the vertical excitation (Franck–Condon) region using normal modes of the ground state.

The central point to recognize in this approach is that the path traced out by gwp 1 plays the role of the traditional reaction path. However, this path is fully coupled to all the other gwp (see Section 2 following). Thus, it explicitly contains the derivative coupling to the other normal modes explicitly. In a traditional reaction path computation, the path may become “curved” by coupling with other normal modes of the same symmetry; however, the effect of the electronic coupling with other nuclear degrees of freedom, which may be of different symmetry and can occur via the derivative coupling, is now included in the approach used here. Further, the path of gwp 1 at a conical intersection is affected not only by the derivative coupling to other normal modes but also by the role of the electronic coherences (see for example refs (5,6)) and these effects are included as well.

We now summarize the central conceptual aspects in this stage. The trajectory followed by the central gwp plays the role of the traditional reaction path. In the classical simulation where the gwps are not coupled and follow classical trajectories, the results will be similar to the traditional reaction path computation except that the path continues through a conical intersection. One could also damp the trajectory obtained in this way.^4^ The 3N-7 normal modes orthogonal to the reaction path become coupled in the usual way when the reaction path curves. However, in the case when the gwp are computed quantum mechanically, there is an additional “curvature” that arises from the coupling of the other gwp due to the derivative coupling as one passes through or near the conical intersection.

Finally, we emphasize that we compute the full effect of the quantum nuclei. However, the reaction path *per se* is taken to be the reaction path followed by the central Gaussian wavepacket (gwp1). How can this idea be probed by experiment? As we shall demonstrate by including a delayed laser pulse in the electronic Schrödinger equation, one may perturb the electronic coherences that occur at the conical intersection.

We will illustrate the protocol described above for the channel 3 radiationless decay of S_1_ benzene (see for example the textbook of Gilbert and Bagshot^27^) as shown in Figure 1. The yield of benzvalene formed from Figure 1c is very low (0.01–0.03)^28^ because the reaction path is diverted toward rearomarization (Figure 1b,d). There is also a competition with intersystem crossing to the triplet^29^ which we will not consider here. In other work,^18^ we have considered the phase mixing^30^ at the conical intersection. In this work, we focus on the reaction path itself so that we always enter the conical intersection region along this path with a very similar phase relationship between the diabatic states.

## 2 Theoretical and Conceptual Development

In a CASSCF computation, one obtains CI expansion coefficients *C*^*k*^(*t*_*i*_) for adiabatic state *k* and orbital rotation coefficients *X*(*t*_*i*_) as a function of time, where time is associated with the step number *i*. The coefficients *C*^*k*^(*t*_*i*_) are obtained by diagonalization of the CI Hamiltonian. In QuEh, rather than diagonalize the Hamiltonian, we introduce a set of coefficients *A*(*t*_*i*_) that mix the adiabatic states propagated as the iterative solution of the time-dependent Schrödinger equation
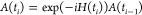
1

The relationship between diagonalization and propagation is well-known
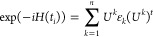
2with
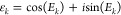
3

The E_k_ values are the eigenvalues associated with the column eigenvectors *U*^*k*^. The coefficients *A*(*t*_*i*_) then determine the gradients and Hessian (see the discussion of second-order Ehrenfest method^15^). The nuclear motion also satisfies the TDSE and uses both the gradients and second derivatives^25,20^ from the electronic structure part. It is formulated in terms of Gaussian wavepackets *g*_*l*_. The important conceptual point is that there is one sequence of Ehrenfest vectors (eq 1) for each gwp *g*_*l*_ as well as a set mixing coefficient gradients and Hessians. We will focus on the reaction path idea so we focus on gwp 1, although this is fully coupled to all the other gwp.

Thus for the nuclear motion, there is a configuration interaction (CI) CI-like representation of Hamiltonian with matrix elements shown in eqs 4 and 5.
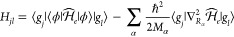
4where
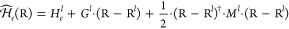
5

The nuclear wave function is expanded in a set of gwps as shown in eq 6.
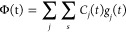
6

The Dirac–Frenkel variational principle^31^ is then used to provide a set of equations for the time propagation of the expansion coefficients and parameters of the gwp (e.g., position and momentum)^32^ To calculate the required matrix elements, we use a quadratic approximation (eq 5) for the Hamiltonian in eq 4, so there is a gradient **G**^*l*^ and Hessian **M**^**l**^ matrix for each gwp *g*_*l*_ which evolve over a time-dependent potential surface and so are in correspondence with an Ehrenfrest trajectory. The coefficient vectors of eq 6 can be analyzed using a population analysis.^33^

The essential point is that the matrix elements in these equations involve an expansion in moments of the Gaussians as well as the gradients and Hessians shown in eq 5. The vector giving the evolution of the Gaussian parameters can be split into a classical part and a purely quantum^24^ term. If the quantum term is omitted, then the nuclei evolve along classical trajectories.

We wish to emphasize that the computed gradients and Hessian in our implementation of Ehrenfest methods (eq 5) use not the average gradient of the states in the superposition; rather, we use the full expressions for the gradient and Hessian from Vacher et al.^15^ These are derived from the general formulation of Almlöf and Taylor.^34^ In contrast, a mean-field approach would involve the average gradient of the two adiabatic states and would not include the cross-interstate mixing terms. We have discussed the effect of these cross-mixing terms in depth elsewhere^17,26,35^

Let us make the connection between the interstate mixing terms and the derivative couplings for clarity. When considering the Hamiltonian in equation 5 expressed in a basis of the adiabatic states with coefficients *A*(*t*) shown in eq 1, the off-diagonal gradients that arise from the mixing of adiabatic states I and II correspond to the derivative couplings (or the interstate coupling vector) and have the form ⟨ψ_I_|∂/∂*q*_*i*_*Ĥ*_*e*_|ψ_II_⟩ where I and II are two adiabatic states and ∂/∂*q*_*i*_ is the gradient operator for each normal mode *i*.

Since the equations for the time propagation of the parameters of the gwp (e.g., position and momentum) involve the gradients and Hessians in eq 5, the equations are fully coupled via the off-diagonal terms in the gradients that are in turn just the derivative couplings.

We conclude this subsection with a brief discussion of how computations are carried out in practice. One starts with an excited state (say S_1_) of a state-averaged computation at an optimized excited state transition state structure with the Hessian computed at that point. The electronic structure and gradients are computed with the second-order Ehrenfest method.^15^ All of the QuEh simulations (one for each Gaussian wavepacket) start initially on the excited state of interest (say S_1_) at the same transition state structure with initial momenta corresponding to excitation of the corresponding normal mode vibration. As one approaches a conical intersection, several states in each Ehrenfest computation become populated (e.g., S_0_ and S_1_). There will be two Ehrenfest computations for each normal mode (positive and negative directions) in addition to the central Gaussian (gwp 1 which we identify with the reaction path and which starts without excitation). We give initial momentum in the normal mode that corresponds to the imaginary frequency. The initial weight of each gwp is zero except for the central wavepacket which has a weight of 1.0. Of course, at each step we have the fully coupled nuclear wave function; however, we identify the reaction path per se with the central Gaussian wavepacket which is fully coupled to the other Gaussian wavepackets.

There is a variant of the above that serves as a reference, namely, classical propagation: In this case, we omit the quantum coupling, keeping all other conditions identical. The key point is that this case omits the effect of the derivative couplings *between* the trajectories as discussed above.

From a practical point of view, the calculations were carried out at 3-SA-CASSCF(6,6)/6–31g(d) level (6 active orbitals and 6 active electrons: S_0_, S_1_, and S_2_) using a development version of Gaussian.^36^ The wave function (CASCI part of the CASSCF computation) was propagated (TDSE) using the Ehrenfest method. In other words, in a CASSCF computation, the CI part of the problem is not diagonalized; here, rather than diagonalization, the wave function is propagated according to the TDSE^15^ (the orbitals themselves satisfy the second order CASSCF equations^37^). All Ehrenfest computations were initiated on the S_1_ state at the optimized transition state Figure 1b with an initial momentum of −1 in mass-frequency scaled units.

Finally, we discuss how to control the electronic coherence at the conical intersection. On the one hand, these coherences are a property of the electronic structure and the electron dynamics occurs when the two states become nearly degenerate (this is in contrast to attochemistry where they are created by a laser pulse). The difficulty is how to characterize these electronic coherences. On the one hand, one may see these coherences in the wave function simply as a superposition of adiabatic states, and we will present our results in this context. But this gives little chemical insight. In another study, we have used some physical properties such as the spin density to follow the electron dynamics.^16^ Here we choose an orthogonal VB representation. The individual VB configurations are quasi diabatic. See the review of Ryabinkin^38^ for a general discussion.

**Figure 2:**
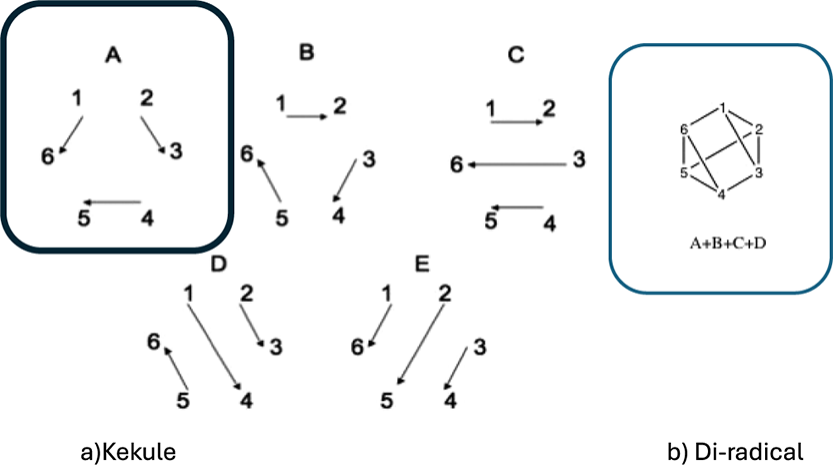
VB quasi-diabatic states for the Benzene Chanel 3 conical intersection. (a) classical VB Rumer structures for benzene: Structure A (boxed) and Structure B (Kekulé), Structure C Structure D Structure E (b) diradical state (S_1_) (obtained from the Structure D by Gram Schmidt orthogonalization). We use structures A (figure a boxed) and b (diradical) as diabatic states.

We have chosen to use the CSF basis in a basis of localized atomic-like orbitals to form this set of states. We focus on CSFs with 6 singly occupied orbitals so we have a set of orthogonal VB-like structures.^39^ The usual full set of nonorthogonal Rumer VB structures is shown in Figure 2a. If these are then Gram-Schmidt orthogonalized (from A to E sequentially), then the fourth structure, which dominates S_1_, is shown in Figure 2b which we refer to as a diradical. These two key structures (A and the diradical) have the same weight at the conical intersection (0.159610, 0.155950) compared with (0.108430, 0.211490) at time zero and (0.234860, 0.022210) at 30 fs. Of course, the remainder of the wave function is made up from combinations of the other Rumer functions and zwitterionic states. But, ′these two states (Figure 2a, Structure A, Kekule′ and Figure 2b, Diradical) dominate and serve as quasi-diabatic states. Thus the electronic coherences satisfy two properties (i) the weights of the adiabatic states are very similar and (ii) we can see oscillations in the diabatic state populations.

## 3 Results and Discussion

We shall focus our discussion on three simulations: (i) classical nuclei, (ii) full QuEh, and (iii) full QuEh with an IR pulse. In comparing i) and ii), we see the effect of the reaction path curvature (Figure 1) when the coupling to the derivative coupling between normal modes is allowed.

### Classical Nuclear Motion

**Figure 3:**
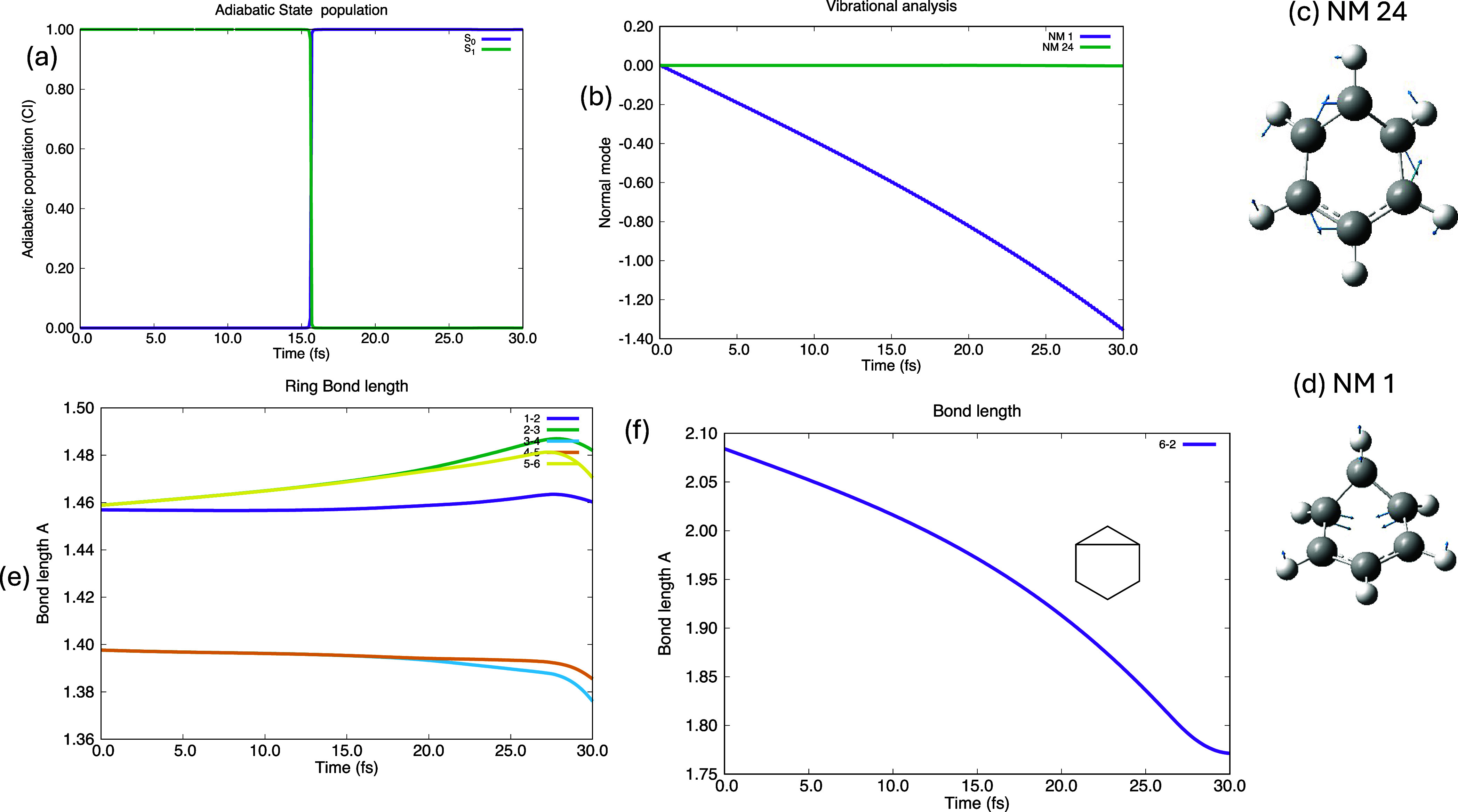
Classical nuclear motion for gwp 1 (the reaction coordinate) (a) the population of S_0_ and S_1_ as a function of time showing decay at the conical intersection at 16 fs. (b) The expectation value of nm 1 (6–2 bond length)and nm 24 (derivative coupling). (c) nm 24. (d) nm 11. (e) Ring bond lengths. (f) 6–2 prefulvene bond length

We use the term classical nuclear motion to denote ignoring the quantum terms in the gwp propagation (i.e., effectively an uncoupled second-order Ehrenfest method). We obtained the picture shown in Figure 3. In Figure 3a we give the populations of S_0_ and S_1_ with time. There is a sharp “clean” transition from S_1_ to S_0_ at about 16 fs. In Figure 3b we give the population of two important normal modes (Figure 3c shows the derivative coupling at nm 24 (nm for normal mode), and Figure 3d shows the reaction path at nm 1). Note that the derivative coupling (nm 24 Figure 3c), leads to a localized Kekulé structure with bonds 1–2, 3–4, and 5–6 shorter than 2–3, 4–5, and 6–1. The reaction path (nm 1 Figure 3d) corresponds to a transannular 6–2 bond length change.

In Figure 3b, we see that only the reaction path normal mode (NM1 Figure 3d) is populated (i.e., changes). In Figures 3e and 3f we give the ring bond lengths and the transannular 6–2 bond length (which is the reaction coordinate nm 1). One can see in Figure 3e,f that the ring bond lengths (Figure 3e) remain almost constant (when the path traced by gwp 1 goes through the conical intersection at 1.96 Å, Figure 3a, toward S_0_ prefulvene at 1.59 Å). The derivative coupling, which is along nm 24 (Figure 3c), does not couple to the reaction path. This is because normal mode 24 has a different symmetry. However, the main point is that with classical motion the gwp is not coupled by terms such as  (in eqs 4 and 5) and the corresponding off-diagonal first and second derivatives. We now proceed to discuss the changes resulting from the derivative coupling in full quantum similation.

### Fully Coupled Nuclear Motion QuEh

**Figure 4:**
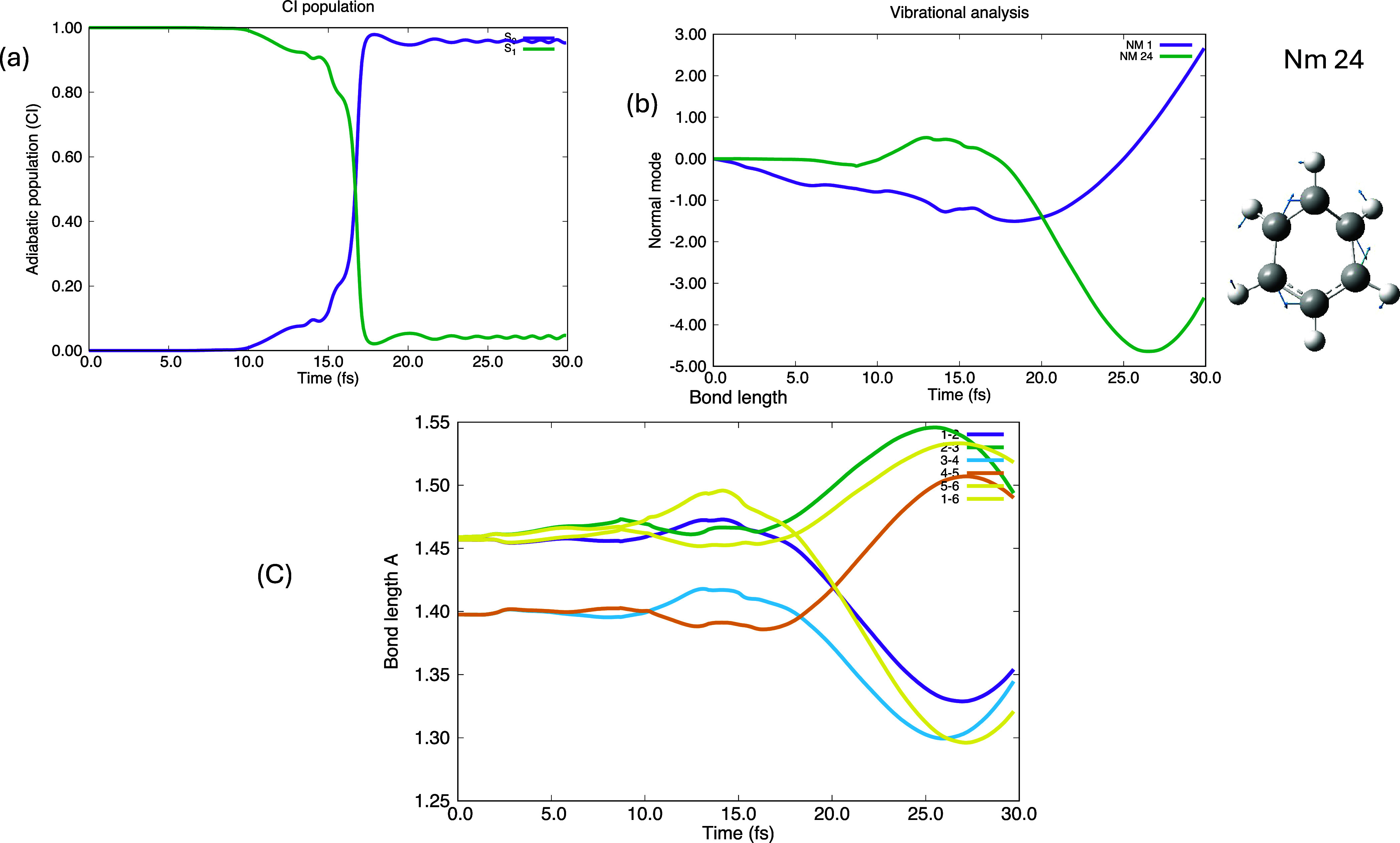
Fully quantum (QuEh) nuclear motion for gwp 1 (the reaction coordinate) (a) the population of S_0_ and S_1_ as a function of time showing decay at the conical intersection at 16 fs. (b) The expectation values of of nm 1 (6–2 bond length) and nm 24 (derivative coupling). (c) Ring bond lengths.

The results for gwp1 in the fully coupled regime are summarized in Figure 4) in the same format as Figure 3 (except that there is no part d because we have this effect in nm 1 in Figure 4b). In Figure 4a, we again show the populations of S_0_ and S_1_ with time. Now the S_0_–S_1_ decay is not instantaneous. In the time period between 10 and 16 fs both the S_0_ and S_1_ are populated, and we have a coherent superposition of the two states. (In attochemistry^19,40,41^ this coherent superposition is created with a laser pulse). This effect comes from the coupling with nm 24. The increasing contribution of the derivative coupling from 10 fs is shown in Figure 4b. This has the effect of diverting the reaction path (see Figure 1), from the (6–2) bond length (Figure 1b–d) along the derivative coupling nm 24 from about 10 fs. One can be seen most clearly in the ring bond lengths, plotted in Figure 4c. It is clear that we have decayed to a localized Kekulé structure with bonds 1–2, 3–4, and 5–6 shorter than 2–3, 4–5 and 6–1 (Figure 4c).

**Figure 5:**
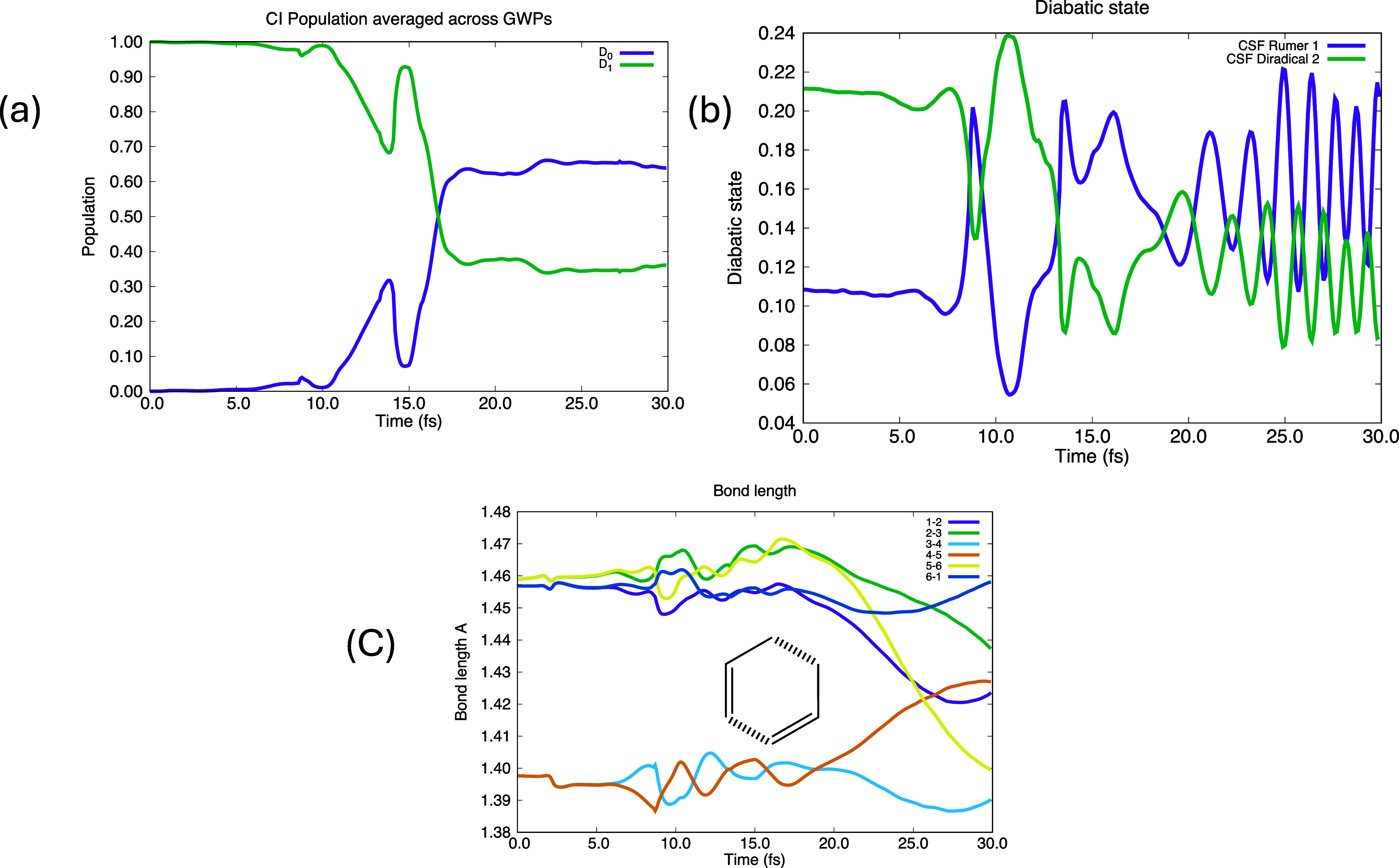
Dynamics parameters computed from the full nuclear wave function in the same representation as Figure 3. (a) Population of S_0_ and S_1_ as a function of time showing partial decay at the conical intersection at 10–16 fs. (b) Diabatic state populations. (c) Ring bond lengths

Let us now elaborate on the fact that decay in Figure 4a is not instantaneous (compare with Figure 3a). This is an effect of the electronic coherences, as shown in Figure 5, where we give results for the complete nuclear wave function. Any property arising from a complete nuclear wave function is similar to the average that one performs in surface hop computations except that the weighting of the results in this case is from quantum chemistry. One can see (Figure 5a) that the surface hop is spread out over 5 fs and the result is propagation on a mixed state (like attochemistry as discussed above but induced by the conical intersection). The corresponding electronic coherence between the diabats shown in Figure 2 is given in Figure 5b. The effect on the ring bond lengths is shown in Figure 5c. We do not see these effects on gwp 1 except that the decay is not instantaneous, because the coupling is not strong enough.

### Fully Coupled Nuclear Motion in the Presence of a Nonresonant IR Pulse^9^ with a Maximum at 10 fs Near the Conical Intersection

**Figure 6:**
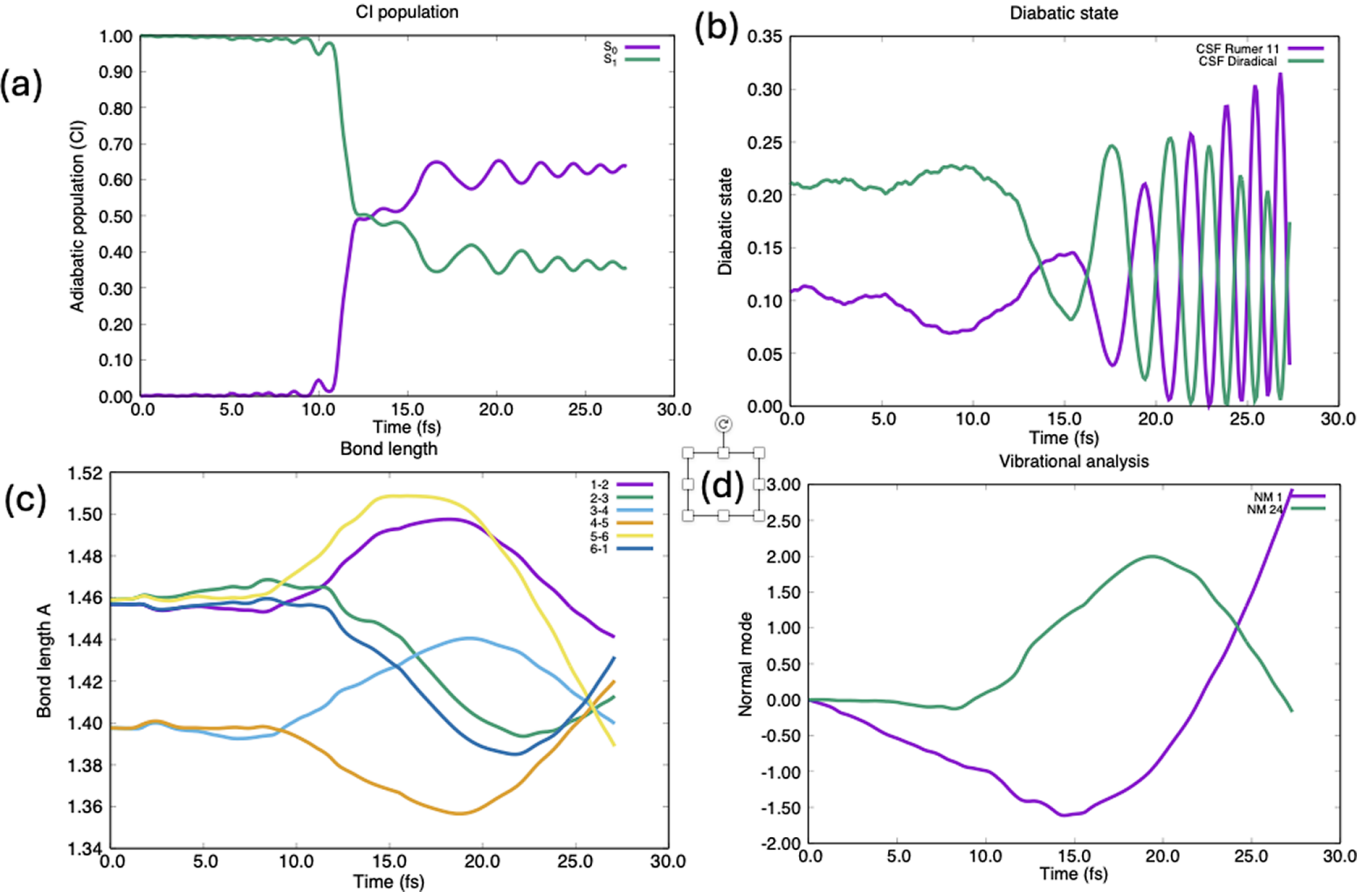
Fully quantum (QuEh) nuclear motion for gwp 1 (the reaction coordinate) with a nonresonant IR pulse. (a) Population of S_0_ and S_1_ as a function of time showing partial decay at the conical intersection at 10 fs. (b) Diabatic state populations. (c) Ring bond lengths. (d) The contribution of nm 1 (6–2 bond length) and nm 24 (the derivative coupling).

We now mix the preceding ideas with a concept from attochemistry by adding a nonresonant 1.4 fs (IR pulse across the molecule) with a maximum at 10 fs, i.e., when the wavepacket is near the conical intersection. This should affect the derivative coupling, nm 24, and enhance the electronic coherences. The results are collected in Figure 6 in a similar format to Figures 3 and 4. On can see in Figure 6a,b that we have now induced electronic coherences with a greatly enhanced contribution of nm 24. There is an effect on the geometry as well. Notice that the Kekulé structure is now formed earlier at 20 fs (Figure 6c) rather than at 27 fs (Figure 4c).

## 4 Conclusions

In this work, we suggest a fully quantum protocol to calculate a chemical reaction path proceeding via a conical intersection, starting at a transition state geometry with initial momentum in the normal mode corresponding to the imaginary frequency. As a result, the reaction path includes the full coupling to other trajectories that have been initiated along the other different normal modes. This reaction path corresponds to the central Gaussian wavepacket (initially unexcited) in the quantum Ehrenfest approach. The results reproduce the curvature of the reaction path induced by the derivative coupling as well as the electronic coherences. The latter effect can be enhanced with an IR pulse.
